# Semi-Empirical Mathematical Modeling, Energy and Exergy Analysis, and Textural Characteristics of Convectively Dried Plantain Banana Slices

**DOI:** 10.3390/foods11182825

**Published:** 2022-09-13

**Authors:** Meenatai G. Kamble, Anurag Singh, Navneet Kumar, Rohini V. Dhenge, Massimiliano Rinaldi, Ajay V. Chinchkar

**Affiliations:** 1 Department of Food Science and Technology, National Institute of Food Technology Entrepreneurship and Management (NIFTEM), Sonipat 131028, India; 2 Food Technology Department, Harcourt Butler Technical University, Nawabganj, Kanpur 208002, India; 3 Department of Processing and Food Engineering, College of Agricultural Engineering and Technology, Anand Agricultural University, Godhara 389001, India; 4 Department of Food and Drug, University of Parma, Parco Area delle Scienze 27/A, 43124 Parma, Italy

**Keywords:** drying kinetics, heat and mass transfer coefficient, activation energy, process energy consumption, hardness

## Abstract

Thin-layer convective drying of plantain banana was performed at four different temperatures from 50 to 80 °C, with slice thicknesses from 2 to 8 mm. The drying curves, fitted to seven different semi-empirical mathematical models, were successfully used to fit experimental data ($R^{2}$ 0.72–0.99). The diffusion approach had better applicability in envisaging the moisture ratio at any time during the drying process, with the maximum correlation value ($R^{2}$ 0.99) and minimum value of $x^{2}$ (2$.5\times 10^{-5}\;\mathrm{to}\;1.5\times 10^{-4})$ and RMSE (5.0 $\times 10^{-3}\;\mathrm{to}\;1.2\times 10^{-2})$. The $D_{eff}$, $h_{m}$, and $E_{a}$ values were calculated on the basis of the experimental data, and overall ranged from $1.11\times 10^{-10}$ to $1.79\times 10^{-9}$ m^2^ s^−1^, $3.17\times 10^{-8}$ to 2.20 $\times 10^{-7}$ m s^−1^ and 13.70 to 18.23 kJ mol^−1^, respectively. The process energy consumption varied from 23.3 to 121.4 kWh kg^−1^. The correlation study showed that the drying temperature had a close correlation with $h_{m}$ value and sample hardness. A significant (*p* < 0.05) increase in hardness of dried plantain banana was observed at 80 °C compared to the other temperatures. Additionally, the sample hardness and process energy consumption were more positively correlated with the thickness of the samples.

## 1. Introduction

Banana is an important fruit crop in many tropical and subtropical areas of India. India is the world’s leading producer of bananas, with a share of around 25% of absolute yield. Andhra Pradesh, Gujarat, Tamil Nadu, Maharashtra, Kerala, Uttar Pradesh, Bihar, and Madhya Pradesh states provide more than 70% of the banana production in India [1]. It constitutes the fourth main staple fruit crop in many economies in developing countries. Among the world’s total fresh fruit produce, 15% of total production is contributed by ~11 × 10^4^ T of banana varieties each year [2]. Plantain banana is a crossbreed and polyploid of two wild, seeded banana species: *Musa acuminata* and *Musa balbisiana.* The composition of banana makes it a good source of carbohydrates (including resistant starch and dietary fiber), phenolic compounds, minerals (potassium, phosphorus, magnesium, and zinc), and vitamins (C, B6, and A), but with low content of protein and fat [3]. The green banana has a low glycemic index, and its consumption could diminish the risk of type II diabetes in the early stage of adulthood [4]. It rapidly deteriorates after harvest due to abiotic factors. In total world production, 33% of bananas are lost because of their climacteric nature [4]. In 2019, the Food and Agriculture Organization estimated that ~2 billion people across the globe did not have the assets required for safer, nutritious, and sufficient food. The United Nations general assembly declared 2021 the international year of fruit and vegetables (IYFV) in order to raise awareness about fruit and vegetables’ vital role in nutrition, health, and food security. In this context, the drying of bananas would be able to solve this issue and reduce wastage, leading to value addition.

Thin-layer hot air convective drying is a commonly used cost-effective preservation method that involves simultaneous heat and mass transfer and which can be properly described using Fick’s diffusion models [5,6]. During drying, the heat energy moves from the surface of the fruit and vegetables, simultaneously enhancing the movement of the moisture inside towards the external surface, resulting in a decline in its moisture content and water activity at a certain level [7,8]. At the same time, it will subsequently minimize the biochemical reaction and microbiological deterioration. Additionally, it helps to minimize the cost of storage, transportation and packaging. Dried plantain bananas are an unexplored ingredient with great potential for application in numerous food products; for example, in flour form, it makes an ideal component of soup mixes, gluten-free cookies, gluten-free biscuits, muffins, baby food mix, and waffles, and makes it possible to modify the taste, flavor, and texture in accordance with consumer preference [3,9].

The information on drying kinetic and critical moisture is fundamental for advancing and controlling the drying process and the quality of the final product. Mathematical modeling is a significant tool that is commonly used to simulate and optimize drying processes. Empirical and semi-empirical mathematical models are widely used to envisage the drying time and drying parameters, and to provide an overview of the drying behaviors of agricultural produce [10]. Several general features influence the drying kinetics of the product, like drying temperature, relative humidity, drying air velocity, and product composition. In addition to the above features, size, shape and surface (morphology) also influence the product’s drying characteristics [10]. This is one of the most energy-intensive operations, due to the high amount of energy consumed in processing compared to the case of fresh produce. Subsequently, there is the need to optimize the drying process, which reduces energy consumption by achieving maximum moisture removal within a short time [11]. Several researchers have studied mathematical modeling in order to better understand the drying behavior of various fruits and vegetables subjected to thin-layer hot-air drying including onion [12], apple [11], lemon [13], banana blossoms [14], mango [15], pumpkin [16], stone apple [17], apple [18], red pepper [19], and green pepper [20].

Moreover, to the best of our knowledge, there is no reported literature which mass transfer properties, process energy consumption, and their compound co-relation are estimated for convectively dried plantain banana slices on the basis of different thicknesses. Additionally, very few authors have studied the mathematical modeling of thin-layer hot air convective drying of bananas at different temperatures and slice thicknesses.

In light of the above limitation, the objective of the present study was to use different semi-empirical mathematical models to predict the moisture ratio (*MR*) as a function of time period. Moreover, effective moisture diffusivity $\left(D_{eff}\right)$, activation energy $\left(E_{a}\right)$, mass transfer properties $(h_{m}),$ process energy consumption, and their compound relation, were determined during the drying process. Additionally, the effect of drying on the textural properties (hardness) of plantain banana slices was studied.

## 2. Materials and Methods

### 2.1. Collection of Raw Material

Fully mature green plantain banana bunches with angularity were hand harvested around 110–120 days after flowering, and were obtained from the Dinesh farm near the district of Sonepat (Haryana, India) in January 2020. The banana fruit bunches were cleaned under running tap water, and then the fruit clusters were separated from the banana bunches and sanitized with sodium hypochlorite (NaOCl) (0.1 g L^−1^) solution to expel the microbial burden from the fruit [21].

### 2.2. Drying Experiment

The green plantain banana fruits, which possessed an initial moisture content of 598.12 ± 13.97% (dry basis), were peeled using a hand peeler. An automated slicer was used to slice the peeled banana into slices of four different thicknesses (2, 4, 6, and 8 mm). Convective tray drying was performed using a laboratory tray dryer (Macro Scientific works Pvt Ltd., Delhi, India) at four different temperatures (50, 60, 70, and 80 °C) with a constant air velocity of 1.5 m s^−1^. All of the distinct banana slices with a weight of 0.88 kg was placed in a single layer on a perforated tray and kept in a dryer for drying. The loss in weight was recorded for each sample, taking out every sample from the dryer at intervals of 10 min during the first 1 h, every 20 min during the second 1 h, every 30 min for the next 1 h, and then after every 1 h until the weight of the banana slices became constant. The weight loss was used to calculate the moisture content of the slices after drying for a certain period of time. The weights of the slices were measured in triplicate using a digital weighing balance (BSA224S-CW, Sartorius Company, Göttingen, Germany; Accuracy = 0.1 mg).

### 2.3. Determination of Moisture Content, Moisture Ratio, and Drying Rate

The moisture content of the banana slices was determined by desiccating sample moisture in a hot air oven at 105 °C to achieve a constant sample weight, and was repeated three subsequent times, as per the AOAC [22] procedure. The moisture ratio (*MR*) and drying rate (*DR*) of the plantain banana slices were calculated using Equations (2) and (3) [23].

(1) $$MR=\frac{\;M_{t}-M_{e}\;}{M_{0}-M_{e}}$$

where $M_{t}$ is the moisture content at any time *t*, $M_{e}$ is the equilibrium moisture content, and $M_{0}$ is the initial moisture content of the sample. The initial moisture content and moisture content of the plantain banana slice at any time point is relatively higher than the equilibrium moisture content $\left(M_{e}\right)$ value of the plantain banana slice. Thus, the equation can be simplified as shown in Equation (2) [24].

(2) $$MR=\frac{M_{t}}{M_{0}}$$

The *DR* of the plantain banana slice was determined under different drying conditions using Equation (3).

(3) $$DR=\frac{M_{t}-M_{t+\Delta t}}{\Delta t}$$

where $M_{t}$ and $M_{t+\Delta t}$ are the moisture content at any given instant *t* and *t* + Δ*t* (kg of water and kg of dry matter^−1^), respectively. Δ*t* is the time difference between two consecutive measurements.

### 2.4. Fitting of Semi-Empirical Mathematical Models

Semi-empirical models typically involve a combination of theory and measurement in order to generalize the design and simplify the results according to observations. The drying data were fitted to seven semi-empirical models obtained on the basis of Fick’s second law of diffusion or Newton’s law of cooling (Table 1). Non-linear regression analysis was used to analyze the drying data and to determine the process-dependent parameters of the model. The drying temperature, drying time, relative humidity, initial moisture content, sample thickness, and morphology are various factors that may influence model parameters. The best model choice for the drying of plantain banana can be distinguished based on three evaluation criteria: the highest coefficient of determination ($R^{2})$, reduced root mean square error (RMSE), and chi-square ($x^{2}$) analysis. The degree of variance between the experimental and predicted values is represented by $x^{2}$ and RMSE. The value of RMSE and $x^{2}$ are inversely proportional to the level of accuracy of the model fit. Estimation of $R^{2}$, RMSE and reduced $x^{2}$ are mathematically expressed in Equations (4)–(6).

(4) $$R^{2}=\frac{\sum_{i=1}^{n}{\left(Y_{i}-{\hat{Y}}_{i}\right)}^{2}}{\sum_{i=1}^{n}{\left(Y_{i}-{\bar{Y}}_{i}\right)}^{2}}$$

(5) $$\mathrm{RMSE}={\left[\frac{1}{N}\sum_{I=1}^{N}{\left(Y_{i}-{\bar{Y}}_{i}\right)}^{2}\right]}^{\frac{1}{2}}$$

(6) $$x^{2}=\overset{n}{\underset{i=1}{\sum }}\frac{{\left(Y_{i}-{\bar{Y}}_{i}\right)}^{2}}{\bar{Y_{i}}}$$

where *N* is the number of observations, and Y and $\bar{Y}$ are the *i*th experimental value and *i*th model-predicted value.

### 2.5. Determination of Effective Moisture Diffusivity

Effective moisture diffusivity is a key phenomenon in the drying process that can be used to determine the rate of movement of water by means of the diffusion of liquid/vapor. The diffusion phenomenon is able to represent the movement of moisture within hygroscopic solid during the falling rate period in accordance with Fick’s second law (Equation (7)) [6]. The diffusion coefficients are considered constant with infinite slab geometry and uniform initial moisture distribution of plantain banana slices.

(7) $$MR=\frac{M_{t}-M_{e}\;}{M_{0}-M_{e}}=\frac{8}{\pi^{2}}\;\overset{\infty }{\underset{n=0}{\sum }}\frac{1}{{\left(2n+1\right)}^{2}}exp\left(-\frac{\left(2n+1\right)\times \pi^{2}\times D_{eff}\times t}{4\times L^{2}}\right)$$

where $D_{eff}$ is the effective moisture diffusivity coefficient (m^2^ s^−1^), *L* is half the thickness of a plantain banana slice (m), *t* is time (s), and *n* is a positive integer (*n* = 0). Thus, for a long drying period, it can be simplified to Equation (8) from Equation (7) [25]. The constant thickness of slices was assumed to have a larger surface area on the cut slices than the peripheral area, resulting in uniform moisture distribution, constant diffusivity, and negligible external resistance and shrinkage [25].

(8) $$MR=\frac{8\;}{\pi^{2}}exp\left(-\frac{\pi^{2}\times D_{eff}\times t}{4\times L^{2}}\right)$$

### 2.6. Determination of Activation Energy

The activation energy $(E_{a}$) describes the association between effective moisture diffusivity and the air temperature of the dryer. It is typically depicted under Arrhenius-type conditions with the aim of investigating the impact of temperature on effective moisture diffusivity, and can be calculated using Equation (9).

(9) $$D_{eff}=D_{0}\;exp\left(-\frac{E_{a}}{R\left(T+273.15\right)}\right)$$

where $D_{0}$ is a pre-exponential factor of the Arrhenius equation (m^2^ s^−1^), $E_{a}$ is the activation energy (kJ mol^−1^), *R* is the universal gas constant (kJ mol^−1^ K^−1^), and *T* is the temperature (°C).

### 2.7. Mass Transfer Properties

The biot number ($B_{i})$ and convective mass transfer coefficient ($h_{m}$) are the mass transfer properties. The $B_{i}$ number is a dimensionless quantity representing the thermal resistance to moisture diffusion inside and the product’s surface. It is calculated by establishing a relation between the $B_{i}$ number and the dimensionless Dincer number ($D_{i}$), and can be conveyed by Equation (10) [26].

(10) $$Bi=\frac{24.848}{Di^{0.375}}$$

$D_{i}$ was calculated using Equation (11), and represents the effect of the flow velocity of the drying fluid and the drying coefficient of the produce [27].

(11) $$D_{i}=\frac{\nu }{k\times L}$$

where *ν* is the drying air velocity (m s^−1^), *L* is the thickness (m), and *k* is the drying constant.

(12) $$h_{m}=\frac{\;B_{i}\times D_{eff}}{L}$$

$h_{m}$ and $D_{eff}$ are associated with the dimensionless number $B_{i}$ for mass transfer, and were calculated using Equation (12) [28]. This equation is legitimate for values of $B_{i}$ greater than 0.1.

### 2.8. Process Energy Consumption

The energy consumed by an electrical heater during the drying process was calculated using Equation (13) [29].

(13) $$E_{T}=A\times v\times \rho_{a}\times C_{a\;}\times \Delta T\times t$$

where *A* is the tray area (m^2^), v is the airflow rate (m s^−1^), *t* is drying time in (h), and Δ*T* is the temperature difference (K). $\rho_{a}$ and $C_{a}$ are the air density (kg m^−3^) and specific heat of the air (kJ kg^−1^ K^−1^), respectively. Drying was carried out at atmospheric pressure, and air density ($\rho_{a}$) at a specific temperature was calculated as expressed in Equation (14) [11]. Specific heat ($C_{a}$) was calculated using Equation (15).

(14) $$\rho_{a}=\frac{101.325}{0.287T_{abs}}$$

(15) $$C_{a}=1.04841-\frac{3.83719T_{abs}}{10^{4}}+\frac{9.45378T_{abs}^{2}}{10^{7}}-\frac{5.49031T_{abs}^{3}}{10^{10}}+\frac{7.92981T_{abs}^{4}}{10^{14}}$$

where $T_{abs}$ is the absolute temperature (K).

The specific energy ($E_{s}$) required for the drying of one kilogram of the sample was determined by using Equation (16) and is expressed as kWh kg^−1^ [29].

(16) $$E_{s}=\frac{E_{T}}{W_{i}}$$

where $E_{T}$ is the total energy required for drying (kWh), and $W_{i}$ is the initial weight of the sample (kg).

### 2.9. Texture Analysis

The texture properties of the plantain banana chips were measured using a texture analyzer (TA-XT plus Texture analyzer, Stable Micro Systems, Godalming, UK) in terms of hardness. The texture analyzer was equipped with a P/2 cylindrical probe loaded with a 30 kg load cell. The force was applied to the banana chips in order to fracture the sample at a pre-test speed of 1.0 mm s^−1^, a test speed of 1.0 mm s^−1^, and a post-test speed of 10.0 mm s^−1^. The hardness of each sample was measured as the peak force in the force deformation curve. Each sample was independently analyzed six times at room temperature [30].

### 2.10. Statistical Analysis

The analysis of variance (ANOVA) was performed for data analysis, and the least significant difference (*p* < 0.05) was calculated using Duncan’s test. The Origin Pro statistical software (pro-2021, Origin Lab Corporation, Northampton, MA, USA) was used to fit the semi-empirical mathematical models. The model coefficients and constants were obtained using the F-test, significant at *p* < 0.05. Furthermore, principal component analysis (PCA) and dendrogram analysis were performed to determine the compound correlation among the different tray-dried samples.

## 3. Results and Discussion

### 3.1. Drying Characteristics

**Figure 1:**
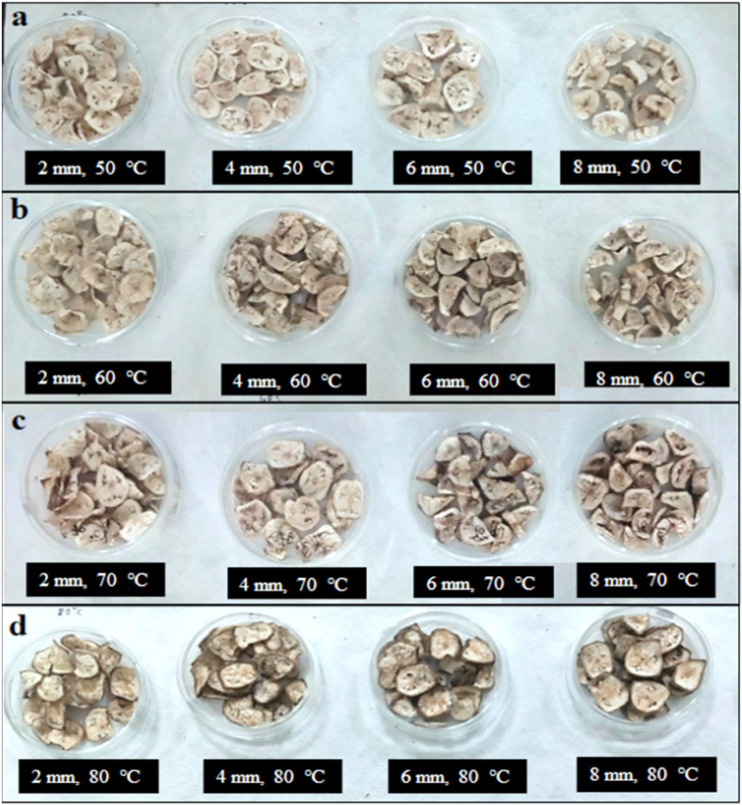
Convective hot-air-dried plantain banana slices with thicknesses of 2, 4, 6, and 8 mm dried at 50 °C (**a**), 60 °C (**b**), 70 °C (**c**) and 80 °C (**d**).

**Figure 2:**
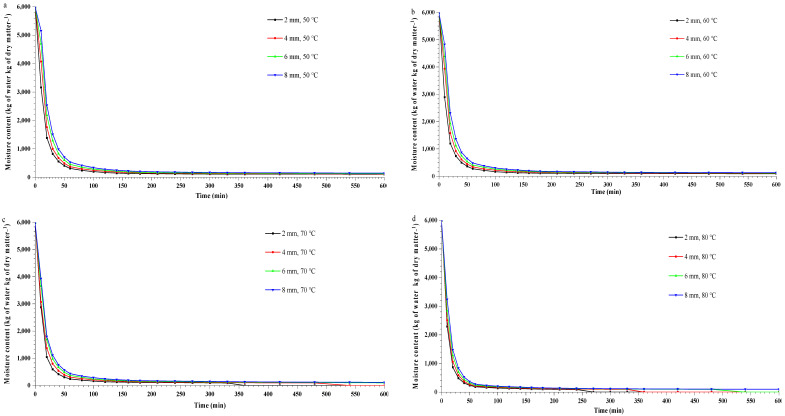

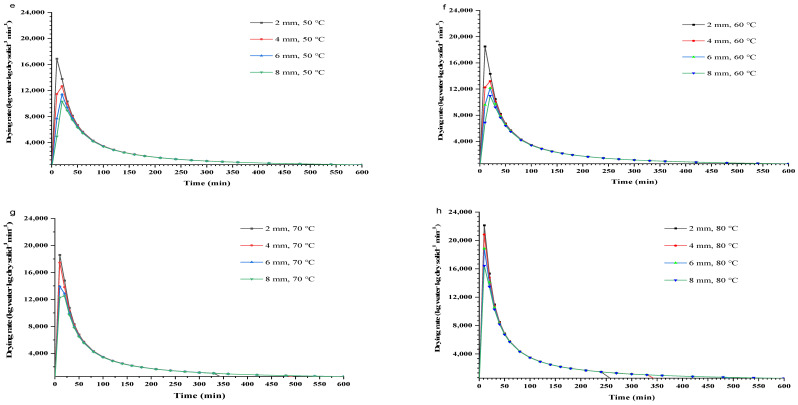
Variation in moisture content vs. drying time (**a**–**d**) and drying rate vs. drying time (**e**–**h**) for plantain banana slices.

The dehydrated plantain banana slices with thicknesses of 2, 4, 6 and 8 mm, dried at four different temperatures (50–80 °C), are shown in Figure 1a–d. The variations in moisture content and drying rate (*DR*) with drying time for plantain bananas were investigated at four different drying temperatures (50, 60, 70, and 80 °C) and thicknesses (2, 4, 6 and 8 mm) (Figure 2a–h). It was observed that the dehydration of banana slices could be achieved at a constant temperature with varied thickness by hot air-drying. With increasing drying temperature, the movement of the water molecules increases and evaporates rapidly from the minimum thickness of the food surface. The final moisture content obtained at four different temperatures and thicknesses was found to be in the range of 11.89–12.33% (dry basis). A significant (*p* < 0.05) reduction in drying time was observed when drying the 2, 4, 6 and 8 mm banana slices at four different drying temperatures (50–80 °C). For the slice with a thickness of 2 mm, the drying time was decreased by 66.7% at 80 °C compared to the time required at 50 °C, as was expected. On the other hand, for the slices with thicknesses of 4, 6 and 8 mm, the drying time decreased by 62.5, 66.7, and 71.9%, respectively, when dried at 80 °C compared to when dried at 50 °C. A similar finding was reported by Kabiru et al. [15] and Sharabiani et al. [31] for the drying of apple slices and mango.

On the basis of the drying curves, it was observed that the falling rate period for the 2 mm slice was 1 h, with 0.24 kg of water loss per kg of dry matter at 50 °C (Figure 2a). Meanwhile, the falling rate period at 50 °C for the slices with thicknesses of 4, 6 and 8 mm was sustained for 1.33, 1.66 and 2 h, respectively, and decreased the initial moisture content by 0.23, 0.23, and 0.22 kg of water per kg of dry matter, respectively. The constant *DR* was trailed by the falling rate of drying, which consequently decreased with increasing drying time, as shown in Figure 2e–h. A similar pattern of constant *DR* followed by falling rate was obtained for 2, 4, 6, and 8 mm thick banana slices dried at 60, 70, and 80 °C, as shown in the drying curves presented in Figure 2f–h. The thickness of the plantain banana slices had a significant (*p* < 0.05) effect on moisture content and drying time. Srikanth et al. [32] and Jabeen et al. [33] stated that the decreased kinetic energy of water molecules resulted in a slower *DR* with increasing sample slice thickness. The same trend was noticed on plantain banana slices dried at 60, 70, and 80 °C with varied slice thicknesses (2 to 8 mm) (Figure 2). The faster reduction in moisture content during the initial drying phase could be attributed to the ambient availability of surface moisture and its subsequent diffusion to keep up with the constant *DR* during drying at various temperatures and product thicknesses [34]. Ojediran et al. [35] noted that as the drying process progressed, the diffusion of moisture from the core to the surface of each particle became the limiting factor, greatly lowering the *DR* value due to the bound water of the samples. For the overall drying process, the falling rate period was observed as an eventual outcome of the mass transfer-controlled process.

### 3.2. Model Fitting on Drying Curve

The *MR* data were obtained at four different drying temperatures (50, 60, 70 and 80 °C) with constant thicknesses of 2, 4, 6 and 8 mm. On the basis of the experiments, the obtained drying data were fitted to seven semi-empirical mathematical models to envisage the drying behavior (Table 1). The drying curve model constant and coefficients were evaluated. The results were analyzed in terms of the highest $R^{2}$ value, lowest reduced $x^{2}$, and RMSE for all the drying conditions to assess the applicability of the better fit model for different drying curves, represented in Table 2. The F-values of all models were seen as significant (*p* < 0.05). From all the experiments, all models $R^{2}$, $x^{2}$, and RMSE values varied in between the range of 0.72 to 0.99, $2.5\times 10^{-5}$ to $1.0\times 10^{-2}$ and $5.0\times 10^{-3}$ to $1.0\times 10^{-1}$, respectively. The exception of the Verma model showed non-fit to the experimental data of the 2 mm banana slice dried at 60 and 80 °C, respectively. Whereas modified page, page, diffusion approach, and logarithmic models quite fit well to the experimental data dried at varied temperatures and thicknesses, and it showed the $R^{2}>$0.91. Likewise, Henderson and Pabis, and Newton/Lewis models were observed to be the best fit with the $R^{2}$ > 0.92 for experimental data of 2 mm plantain banana slices dried at 70 and 80 °C, respectively. The Verma model was found to be the best fit for the experimental data of banana slices dried at 70 °C having 2 mm thickness and 80 °C, having 6 mm thickness, respectively, showing the $R^{2}$ > 0.92. Among all the mathematical drying models, the diffusion approach model was observed to be better fitted to all experimental drying curve data obtained from different drying conditions, which was selected based on the maximum $R^{2}$ 0.99 and minimum $x^{2}\;\left(2.5\times 10^{-5}\;\mathrm{to}\;1.5\times 10^{-4}\right)$ and RMSE value $(5.0\times 10^{-3}\;\mathrm{to}\;1.2\times 10^{-2}$).

**Figure 3:**
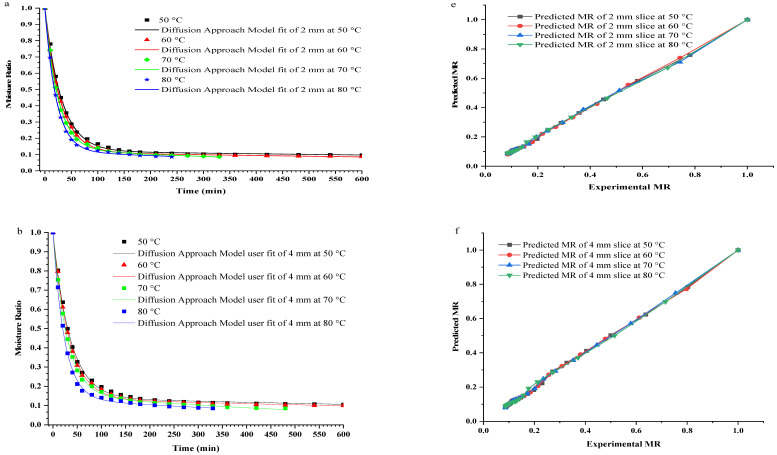

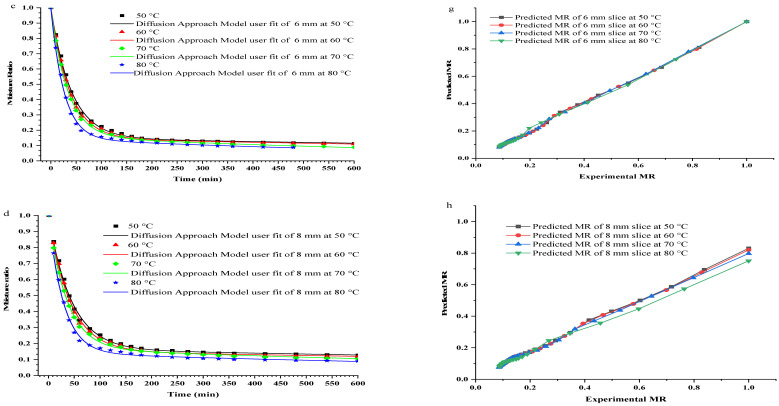
The moisture ratio (*MR*) vs. drying time (**a**–**d**) and experimental moisture ratio (*MR*) vs. predicted moisture ratio (*MR*) (**e**–**h**) for plantain banana slices at different drying temperatures and thickness by diffusion approach.

The most appropriate fitted drying curve models (diffusion approach model) were validated by plotting the experimental *MR* and predicted *MR* of bananas dried at different temperatures with constant thickness against time, represented in Figure 3a–d.

The fit agreement was generated between the predicted and experimental *MR* at different drying temperatures with constant thickness using the diffusion approach model (Figure 3e–h). Henceforth, it could be contemplated that the diffusion approach model was seen to be satisfactory in addressing the drying behavior of banana slices at different temperatures and constant thicknesses. This assessment is in concurrence with a study conducted by Yaldýz & Ertekýn [20]. They studied the hot air drying of green pepper at 50–80 °C and showed that the diffusion approach model best fit the experimental *MR* data. Additionally, red pepper was hot air-dried at a temperature of 55–70 °C showed the diffusion approach model is ideal to foresee the drying of red pepper [19]. Interestingly, the diffusion approach and logarithmic model were found to be the best fit for hot air-dried banana slices (4 mm in thickness) at 60 and 70 °C, respectively, with the highest $R^{2}$ 0.99 and 0.99, respectively [36].

### 3.3. Effective Moisture Diffusivity

The effective moisture diffusivity during drying is a complex process involving various diffusion parameters, such as liquid, molecular, vapor, and hydrodynamic diffusion, to describe the characteristic movement of the inherent moisture of food [37]. The effective moisture diffusivity usually relies on drying temperature, air velocity, relative humidity, and sample thickness [38]. The $D_{eff}$ values for the drying of plantain banana slices under various drying temperatures and thicknesses were obtained within a range of $1.11\times 10^{-10}$ to $1.79\times 10^{-9}$ m^2^ s^−1^, with $R^{2}$ values ranging from 0.79 to 0.90, as shown in Table 3. These results are in agreement with the $D_{eff}$ values of food material ($10^{-12}–10^{-8}$ m^2^ s^−1^) reported by Zogzas et al. [38]. The $D_{eff}$ values of the 2, 4, 6, and 8 mm banana slices dried at a constant temperature of 50 °C were found to be $1.11\times 10^{-10},$ $3.66\times 10^{-10}$, $6.89\times 10^{-10}$ and $1.04\times 10^{-9}$ m^2^ s^−1^, respectively, with the highest $R^{2}$ being 0.79–0.85, and the lowest values of $x^{2}$ $(7.7\times 10^{-3}$ to $9.7\times 10^{-3})$ and RMSE $(8.7\times 10^{-2}$ to $9.8\times 10^{-2}$). The increase in the effective moisture diffusivity of banana slices was observed upon increasing the thickness of the sample (2–8 mm) at 50 °C. In the same way, the same effective moisture diffusivity trend was noticed for banana slices with thicknesses of 2, 4, 6, and 8 mm dried at 60, 70, and 80 °C, which was found to be in the range of $1.25\times 10^{-10}$ to $1.15\times 10^{-9}$ m^2^ s^−1^, $1.41\times 10^{-10}$ to $1.28\times 10^{-9}$ m^2^ s^−1^ and $1.70\times 10^{-10}$ to $1.79\times 10^{-9}$ m^2^ s^−1^, respectively, as shown in Table 3. In the same way, an increase in the effective moisture diffusivity pattern was seen for banana slices dried at 50, 60, 70, and 80 °C with a constant slice thickness of 2, 4, 6, and 8 mm (Table 3). The hot air increases the heat energy and the activity of water molecules resulting from the increase in vapor pressure inside the food samples, prompting an enhancement of effective moisture diffusivity toward the outer surface [11]. Therefore, the $D_{eff}$ value of the banana slice was seen to increase with increasing drying temperature and banana slice thickness (Table 3). Additionally, Ojediran et al. [35] noted that the $D_{eff}$ of yam slices increases with increasing air velocity (0.5, 1, and 1.5 m s^−1^), temperature, (50, 60 and 70 °C), and thickness (3, 6 and 9 mm), which was acquired in ranges from $6.4\times 10^{-9}$ to $1.6\times 10^{-7}$ m^2^ s^−1^. Moreover, Kumar et al. [23] evaluated the $D_{eff}$ value of thin-layer-dried banana slices (4 mm) at 45–65 °C, and increases from $3.79\times 10^{-8}$ to $6.23\times 10^{-8}$ m^2^ s^−1^ were observed with increasing drying temperature. The $D_{eff}$ value of hot air-dried apple slices at 50, 60, and 70 °C was seen to be in the range of $6.75\times 10^{-10}$ to $1.28\times 10^{-9}$ m^2^ s^−1^ [11].

These differences in the effective moisture diffusivity of agricultural produce might be because of the diversity in the nature of biomaterials, composition, dimensions, drying velocity, drying temperature, and moisture content, as well as physical or chemical pretreatment conditions [39].

### 3.4. Activation Energy

The commencement of the reaction (moisture diffusion) from the inside to the outside of plantain banana slices requires energy, which is expressed as activation energy $\left(E_{a}\right)$. This is the energy needed to achieve effective moisture diffusivity. Higher values of $D_{eff}$ are dependent on temperature and thickness, and enhance the $E_{a}$ and promote moisture movement. $E_{a}$ was calculated using the Arrhenius equation, and was found to be in the range of 13.70 to 18.23 kJ mol^−1^ for banana slices dried at four different temperatures (50, 60, 70, and 80 °C) and thicknesses (2, 4, 6 and 8 mm), with highest $R^{2}$ value ranging from 0.89 to 0.98, and the lowest $x^{2}\;(1.93\times 10^{-23}$ to $1.7\times 10^{-20})$ and RMSE $(4.39\times 10^{-12}$ to $1.30\times 10^{-10}),$ as shown in Table 3. Values of $E_{a}$ for banana slices dried at different temperatures were obtained as 13.70, 15.82, 16.93, and 18.23 kJ mol^−1^, respectively, for slice thicknesses of 2, 4, 6, and 8 mm. The obtained results are within the range of those published by various researchers with respect to several food materials (12.7–110 kJ mol^−1^) [40]. Therefore, it can be concluded that the $E_{a}$ value is also dependent on sample thickness. Several researchers have reported the $E_{a}$ values for the drying of various types of agriculture produce; for example, the $E_{a}$ value of *Dioscorea rotundata* and *Dioscorea alata* increased insignificantly (*p* > 0.05) from 41.75 to 72.47 kJ mol^−1^ and 25.25 to 46.46 kJ mol^−1^, respectively, during drying at different temperatures (50–80 °C) and thicknesses (10–30 mm) [39]. In the same way, the $E_{a}$ value of yam sliced was found to be 10.59–54.93 kJ mol^−1^, which increased with increasing air velocity (0.5–1.5 m s^−1^) but fluctuated with air temperature and thickness [35]. John et al. [14] evaluated the $E_{a}$ value of hot air-dried banana blossoms at temperatures of 40–60 °C, and found that it was 500 kJ mol^−1^. Likewise, the $E_{a}$ value of apple slices dried at three different temperatures—50, 60, and 70 °C —and air velocities—1, 1.5, and 2 m s^−1^—were 17.77, 19.75, and 25.41 kJ mol^−1^ [11]. Moreover, the $E_{a}$ value of melon slices dried at four different temperatures—40, 50, 60, and 70 °C—with constant thicknesses of 2, 4, and 6 mm, respectively, were found to be 45.24 to 51.30 kJ mol^−1^ [41]. Thus, various drying parameters, such as drying temperature, air velocity, sample thickness, and moisture diffusivity, significantly affect $E_{a}$ value.

### 3.5. Mass Transfer Properties

The mass transfer coefficient ($h_{m}$) increased with decreasing moisture content and increasing drying temperature and thickness. The value of $h_{m}$ increased from $3.16\times 10^{-8}$ to $2.20\times 10^{-7}$ m s^−1^ with increasing drying air temperature (50–80 °C) and slice thickness (2–8 mm), as shown in Table 3. Similarly, Darvishi et al. [41] found that the value of *h_m_* increased from $1.07\times 10^{-9}$ to $10.68\times 10^{-9}$ m s^−1^ in melon slices dried at temperatures of 40, 50, 60, and 70 °C and slice thicknesses of 2, 4, and 6 mm, respectively. In the same way, Guiné et al. [28] assessed the $h_{m}$ of pumpkin slices (5–12.5 mm) dried at 30–70 °C, finding an increase in the value from $0.51\times 10^{-6}$ to $27.8\times 10^{-6}$ m s^−1^, while pear slices dried at 60 and 70 °C were found to exhibit an increase from $3.5\times 10^{-11}$ to $11.2\times 10^{-11}$ m s^−1^. Furthermore, the increase in the value of $h_{m}$ of hot air-dried whole lemon at 50, 60, and 70 °C was found to be $0.3626\times 10^{-6}$, $0.805\times 10^{-6}$ and $3.374\times 10^{-6}$ m h^−1^, respectively [13]. The increase in the value of $h_{m}$ with increasing drying temperature and slice thickness due to surface hardening results in an increase in the diffusion of moisture toward the outer surface [41].

### 3.6. Process Energy Consumption

**Figure 4:**
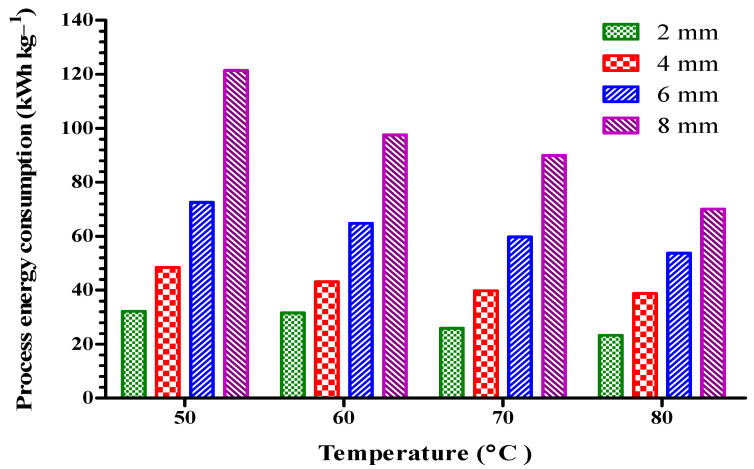
Process energy consumption of plantain banana slices during convective hot air drying at different temperatures and thicknesses.

The process energy consumption required for the drying of plantain banana at different temperatures and thicknesses was found to be between 23.26 and 121.44 kWh kg^−1^, as shown in Figure 4. The maximum reductions in the process energy consumption of 2 mm thick dried plantain banana were 1.81, 19.63, and 27.61%, respectively, at temperatures of 50 to 60, 70 and 80 °C. A similar trend was found for plantain bananas with thicknesses of 4, 6, and 8 mm dried at four different temperatures, as shown in Figure 4. However, the maximum increases in the energy consumption of dried plantain banana at the four different thicknesses of 2, 4, 6, and 8 mm were found to be from 32.13 to 121.44, 31.55 to 97.51, 25.82 to 89.92, and 23.26 to 70.08 kWh kg^−1^, at temperatures of 50, 60, 70 and 80 °C, respectively. The increase in drying temperature resulted in a decline in drying time, and had a more significant effect on the process energy requirement of the dried sample [29]. The process energy consumption of dried samples was more influenced by air velocity, drying time, and drying temperature [11,30,40].

### 3.7. Texture

The textural properties, characterized in terms of hardness, are the force necessary to determine the capacity of a material to resist deformation. The variation in the hardness of plantain banana slices of different thicknesses dried at different temperatures is shown in Table 3. Insignificant (*p* > 0.05) increases in the hardness of dried plantain banana at 50, 60, and 70 °C were observed, from 15.25 to 23.75, 26.56 to 37.02, 38.23 to 45.76, and 39.8 to 5.36 N for the constant thicknesses of 2, 4, 6, 8 mm, respectively. The significant (*p* < 0.05) increase in the hardness of plantain banana slices was found to be at 80 °C, with varied thicknesses of 2, 4, 6, and 8 mm, respectively (Table 3). The significant (*p* < 0.05) differences in the hardness of dried plantain banana with four different thicknesses of 2, 4, 6, and 8 mm were found to be 15.25 to 39.8, 17.54 to 41.65, 23.75 to 50.36, and 31.84 to 71.41 N at constant drying temperatures of 40, 50, 60 and 70 °C, respectively. The water vapor expands the cellular wall when the hot air comes into contact with the fruit surface. This creates large pores within the center of the material, showing the highest hardness with dense structure compared to when dried at the lowest drying temperature [42]. Conversely, at the minimum drying air temperature, hot air moved from the surface to the core at a slow and constant speed, causing insignificant microstructural changes and leading to this sample having the lowest hardness [43]. Similarly, Kumar et al. [23] observed an increase in the hardness of a thin layer of dried ripe banana from 6 to 10 N at temperatures of 110–140 °C. Additionally, Macedo et al. [44] reported that the rapid loss of moisture content with increasing air drying temperature results in greater physical damage to food material, leading to an increase in sample hardness.

### 3.8. Compound Correlation Study

**Figure 5:**
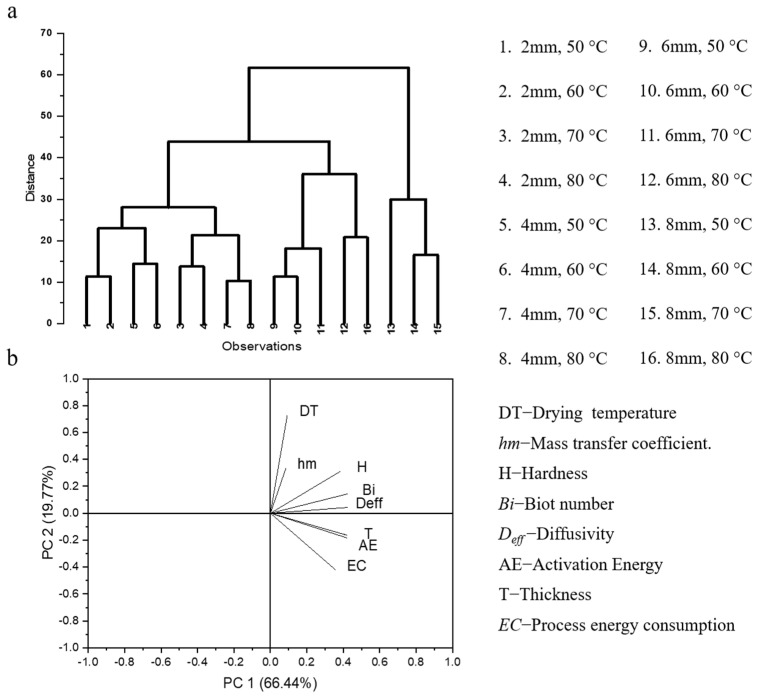
(**a**) Dendrogram representing compound correlation among different samples, (**b**) PCA loading plot representing correlation among various drying characteristics.

A compound correlation among the different samples can be observed from the dendrogram considering various independent and dependent parameters presented in Figure 5. The samples with lower thicknesses (2 and 4 mm) and at lower temperatures (50 and 60 °C) are closely correlated, and have similar drying characteristics. The samples with greater thicknesses (6 and 8 mm) also possess similar characteristics at the higher temperature of 80 °C. Overall, the samples can be clustered into four groups, i.e., (i) 1, 2, 5, 6; (ii) 3, 4, 7, 8; (iii) 9, 10, 11, 12, 16; and (iv) 13, 14, 15. The correlation among the various samples is evident, due to the smaller changes in characteristics under similar drying conditions. The lowest energy consumption was observed for the samples dried at 80 °C with a thickness of 2 mm, which also had similar drying characteristics to the sample dried at 70 °C with a thickness of 2 mm. Therefore, a sample thickness of 2 mm should be appropriate for drying plantain banana slices. The correlations among the various properties are presented in Figure 5b, which indicates that drying temperature is closely correlated with the $h_{m}$ value. Process energy consumption is more positively correlated with the thickness of the samples. The hardness of the samples is also positively correlated with drying temperature and thickness, indicating that increasing thickness and drying temperature result in increased hardness of the samples.

## 4. Conclusions

Experimental investigation showed that an increase in temperature and reduced thickness significantly influenced the rate of drying, the moisture content, and the drying time. The entire drying experiment was observed to take place within the falling rate period, suggesting the transfer of water molecules from interior to the outer surface of the material was caused by the phenomenon of internal diffusion. The seven semi-empirical mathematical models used to fit the drying curves, the diffusion approach model was found to most adequately fit the experimental data, meeting statistical criteria such as maximum $R^{2}$, and minimum RMSE and $x^{2}$ on the basis of our analysis. The diffusion approach model was found to be the most suitable for predicting the drying behaviors of banana slices of different thicknesses at different temperatures. The $D_{eff}$ and $h_{m}$ of the banana slices increased with increasing drying temperature (50–80 °C) and thickness (2–8 mm). The $E_{a}$ needed for the diffusion of moisture from the inside to the outside of the plantain banana slice was obtained in the range of 13.70 to 18.23 kJ mol^−1^. The process energy consumption was directly affected by sample thickness and drying temperature. However, it is necessary to understand the changes in chemical composition as a result of drying temperature and thickness before their use. This will be performed in future study.

## 5. Practical Applications

The drying of fruits and vegetables is the primary unit activity on most food processing lines, making the installation of proper drying conditions essential. Traditional drying methods lose nutritional quality due to a sluggish drying rate and inappropriate heating. The results obtained in the current study demonstrate banana drying behavior and drying characteristics, which find their application in the determination of the economic aspects of drying banana slices at an industrial scale. Data from this paper could be useful for designing drying operations for this kind of product on the basis of an estimation of drying time, energy consumption, and qualitive parameters by avoiding expensive and time-consuming experimental tests.

## Figures and Tables

**Table 1:** Selected semi-empirical models used for the plantain banana drying kinetics [10].

Sr. No.	Model Name	Model Equation
1	Modified Page	$\mathrm{MR}=exp\left(-{\left(kt\right)}^{n}\right)$
2	Verma	$\mathrm{MR}=a\;exp\left(-kt\right)+\left(1-a\right)exp\left(-gt\right)$
3	Page	$\mathrm{MR}=exp\left(-{kt}^{n}\right)$
4	Newton/Lewis	$\mathrm{MR}\;=exp\;\left(-kt\right)$
5	Logarithmic	$\mathrm{MR}\;=a\;exp\;\left(-kt\right)+c$
6	Henderson and Pabis	$\mathrm{MR}\;=a\;exp\;\left(-kt\right)$
7	Diffusion approach	$\mathrm{MR}=a\;exp\left(-kt\right)+\left(1-a\right)\;exp\left(-kbt\right)$

**Table 2:** Diffusion approach model constant and coefficients at different drying temperatures and thickness with statistical performance indicators (better fit model).

Model	Temperature (°C)	Thickness (mm)	Model Parameters	Statistical Parameters		
				R^2^	Reduced χ^2^	RMSE
Diffusion approach model	50	2	a = 0.88; k = 0.03; b = 0.01	0.99	$4.9\times 10^{-5}$	$6.9\times 10^{-3}$
		4	a = 0.86; k = 0.0287; b = 0.0163	0.99	$6.7\times 10^{-5}$	$8.2\times 10^{-3}$
		6	a = 0.85; k = 0.0249; b = 0.0174	0.99	$6.9\times 10^{-5}$	$8.3\times 10^{-3}$
		8	a = 0.83; k = 0.0226; b = 0.0186	0.99	$6.3\times 10^{-5}$	$8.0\times 10^{-3}$
	60	2	a = 0.88; k = 0.0347; b = 0.0142	0.99	$2.5\times 10^{-5}$	$5.0\times 10^{-3}$
		4	a = 0.86; k = 0.0304; b = 0.0177	0.99	$6.9\times 10^{-5}$	$8.3\times 10^{-3}$
		6	a = 0.85; k = 0.0269; b = 0.0182	0.99	$6.7\times 10^{-5}$	$8.2\times 10^{-3}$
		8	a = 0.84; k = 0.0238; b = 0.0177	0.99	$7.3\times 10^{-5}$	$8.5\times 10^{-3}$
	70	2	a = 0.87; k = 0.0402; b = 0.0329	0.99	$9.8\times 10^{-5}$	$9.9\times 10^{-3}$
		4	a = 0.84; k = 0.0351; b = 0.0411	0.99	$4.1\times 10^{-5}$	$6.4\times 10^{-3}$
		6	a = 0.84; k = 0.0302; b = 0.0343	0.99	$4.4\times 10^{-5}$	$6.6\times 10^{-3}$
		8	a = 0.83; k = 0.0278; b = 0.0284	0.99	$4.6\times 10^{-5}$	$6.8\times 10^{-3}$
	80	2	a = 0.88; k = 0.0465; b = 0.0292	0.99	$9.7\times 10^{-5}$	$9.8\times 10^{-3}$
		4	a = 0.86; k = 0.0427; b = 0.0323	0.99	$1.0\times 10^{-4}$	$1.0\times 10^{-2}$
		6	a = 0.85; k = 0.0386; b = 0.0291	0.99	$1.3\times 10^{-4}$	$1.1\times 10^{-2}$
		8	a = 0.86; k = 0.0341; b = 0.0233	0.99	$1.5\times 10^{-4}$	$1.2\times 10^{-2}$

**Table 3:** Heat transfer parameter, mass transfer parameter, activation energy, and hardness of convectively hot air-dried plantain banana slices.

Sr. No.	Thickness (mm)	Drying Temperature (°C)	$D_{eff}\;(m^{2}\;s^{-1})$	$B_{i}$	$h_{m}$	$E_{a}\;(\mathrm{kJ}\;{\mathrm{mol}}^{-1})$	Hardness (N)
1	2	50	$1.11\times 10^{-10}$	0.57	$3.17\times 10^{-8}$	13.70	15.25 ± 3.20 ^ax^
2		60	$1.25\times 10^{-10}$	0.59	$3.68\times 10^{-8}$		17.54 ± 5.21 ^ax^
3		70	$1.41\times 10^{-10}$	0.62	$4.38\times 10^{-8}$		23.75 ± 6.58 ^ax^
4		80	$1.70\times 10^{-10}$	0.66	$5.58\times 10^{-8}$		31.84 ± 2.02 ^ay^
5	4	50	$3.66\times 10^{-10}$	0.71	$6.50\times 10^{-8}$	15.82	26.56 ± 11.74 ^abx^
6		60	$4.00\times 10^{-10}$	0.73	$7.26\times 10^{-8}$		35.70 ± 9.40 ^bx^
7		70	$4.47\times 10^{-10}$	0.77	$8.57\times 10^{-8}$		37.02 ± 9.57 ^bx^
8		80	$5.90\times 10^{-10}$	0.82	$1.22\times 10^{-7}$		38.67 ± 6.48 ^ax^
9	6	50	$6.89\times 10^{-10}$	0.78	$9.01\times 10^{-8}$	16.93	38.23 ± 4.06 ^bx^
10		60	$7.60\times 10^{-10}$	0.81	$1.02\times 10^{-7}$		39.95 ± 5.71 ^bx^
11		70	$8.35\times 10^{-10}$	0.84	$1.17\times 10^{-7}$		45.76 ± 4.76 ^bcx^
12		80	$1.14\times 10^{-9}$	0.92	$1.76\times 10^{-7}$		66.13 ± 9.57 ^by^
13	8	50	$1.04\times 10^{-9}$	0.84	$1.10\times 10^{-7}$	18.23	39.8 ± 5.26 ^bcx^
14		60	$1.15\times 10^{-9}$	0.86	$1.24\times 10^{-7}$		41.65 ± 7.30 ^bx^
15		70	$1.28\times 10^{-9}$	0.91	$1.46\times 10^{-7}$		50.36 ± 6.58 ^cx^
16		80	$1.79\times 10^{-9}$	0.98	$2.20\times 10^{-7}$		71.41 ± 2.25 ^by^

Mean ± S.D value with superscript (a, b, c and x, y) showing a significant difference (*p* < 0.05) in sample thickness and drying temperature.

## Data Availability

The data that support the findings in this study are available from the corresponding authors upon reasonable request.
